# Enhanced biosynthesis of arbutin by engineering shikimate pathway in *Pseudomonas chlororaphis* P3

**DOI:** 10.1186/s12934-018-1022-8

**Published:** 2018-11-10

**Authors:** Songwei Wang, Cong Fu, Muhammad Bilal, Hongbo Hu, Wei Wang, Xuehong Zhang

**Affiliations:** 1 State Key Laboratory of Microbial Metabolism, School of Life Sciences and Biotechnology, Shanghai Jiao Tong University, Shanghai, 200240 China; 2 National Experimental, Teaching Center for Life Sciences and Biotechnology, Shanghai Jiao Tong University, Shanghai, 200240 China

**Keywords:** Arbutin, *P. chlororaphis*, Plasmid-free strategy, Metabolic engineering, Shikimate pathway

## Abstract

**Background:**

Arbutin is a plant-derived glycoside with potential antioxidant, antibacterial and anti-inflammatory activities. Currently, it is mainly produced by plant extraction or enzymatic processes, which suffers from expensive processing cost and low product yield. Metabolic engineering of microbes is an increasingly powerful method for the high-level production of valuable biologicals. Since *Pseudomonas chlororaphis* has been widely engineered as a phenazine-producing platform organism due to its well-characterized genetics and physiology, and faster growth rate using glycerol as a renewable carbon source, it can also be engineered as the cell factory using strong shikimate pathway on the basis of synthetic biology.

**Results:**

In this work, a plasmid-free biosynthetic pathway was constructed in *P*. *chlororaphis* P3 for elevated biosynthesis of arbutin from sustainable carbon sources. The arbutin biosynthetic pathway was expressed under the native promoter *P*_*phz*_ using chromosomal integration. Instead of being plasmid and inducer dependent, the metabolic engineering approach used to fine-tune the biosynthetic pathway significantly enhanced the arbutin production with a 22.4-fold increase. On the basis of medium factor optimization and mixed fed-batch fermentation of glucose and 4-hydroxybenzoic acid, the engineered *P. chlororaphis* P3-Ar5 strain led to the highest arbutin production of 6.79 g/L with the productivity of 0.094 g/L/h, with a 54-fold improvement over the initial strain.

**Conclusions:**

The results suggested that the construction of plasmid-free synthetic pathway displays a high potential for improved biosynthesis of arbutin and other shikimate pathway derived biologicals in *P. chlororaphis*.

**Electronic supplementary material:**

The online version of this article (10.1186/s12934-018-1022-8) contains supplementary material, which is available to authorized users.

## Background

Plant-derived secondary metabolic compounds, such as alkaloids, phenols, saponins, and terpenoids, have been widely isolated from plant cells with commercial applications [1]. Owing to their potential antimalarial, anti-tumor, anti-allergy, anti-inflammatory, antibacterial, antioxidant and anti-aging activities [2–4], these biologicals are extensively concerned in the fields of foodstuffs, daily chemicals, and medical treatment. The complexity and diversity of these natural products can be extended by glycosylation, methylation, hydroxylation, and prenylation [5]. Glycosylation reactions mainly catalyzed by glucosyltransferases (Gts) can alter the physicochemical and biological activities of many exogenous compounds. In recent years, glycosylated natural compounds, such as anthocyanins, gastrodin and glucosinolates have been widely studied due to their enormous physiological and pharmaceutical activities [6–8].

Arbutin is a hydroquinone glycoside with great capacity to inhibit melanin formation for skin-lightening efficacy [9]. It is a widely used and effective agent in the medical, healthcare and cosmetic industries thanks to its anti-oxidant, anti-microbial and anti-inflammatory activities [10, 11]. The existing arbutin production is largely relied on chemical routes and plant-based extraction processes, which presents the drawbacks of complex procedures and low product yields. In the past decades, enzymatic catalysis has gained a considerable research attention owing to the identification of various functional glucosyltransferases. For instance, 0.544 g/L arbutin was produced from 49.5 g/L quinol (HQ) when catalyzed by purified dextransucrase [12]. The whole-cell bioconversion with surface-anchored transglucosidase enabled 21 g/L arbutin production from 200 g/L glucose in high cell density of *Escherichia coli* under fed-batch fermentation [13]. Nevertheless, 4.19 g/L arbutin was accumulated in *E. coli* by plasmid-based expression of biosynthetic enzymes using the shikimate pathway under optimized glucose concentration [11]. The biosynthetic pathway of plant-derived arbutin has been well elucidated in *E*. *coli* by evaluating the efficient 4-hydroxybenzoate 1-hydroxylase encoded by *MNX1* and glucosyltransferase encoded by *AS* with high specificity using versatile platform intermediate 4-hydroxybenzoate (4-HBA) as a precursor [11, 14].

*Pseudomonas chlororaphis* P3 is a mutant strain obtained from *P. chlororaphis* HT66 with multiple rounds of chemical mutagenesis and selection, which can produce 4.7-time higher phenazine-1-carboxamide (PCN) (2.10 g/L) than that of the native counterpart. It has been engineered as a platform organism due to the relatively well-characterized physiology and genetics. *P. chlororaphis* displays fast cell growth rate on glycerol, which has become an emerging feedstock for the biosynthesis of value-added chemicals since it is an inevitable by-product. Recently, iTRAQ-based quantitative proteomic analysis unveiled the metabolic capacity of *P*. *chlororaphis* P3 and provided valuable clues to better apprehend the biosynthesis, excretion, and regulation of PCN in the strain [15]. On the basis of known potential gene targets, *P*. *chlororaphis* P3 was selected as an alternative source to construct high-yielding chorismate derivatives strains with native promoter. For industrial-scale production of arbutin, plasmid-free strategy offers economic and environmental benefits thus presenting a strong potential to compete with the plasmid-based expression.

**Fig. 1:**
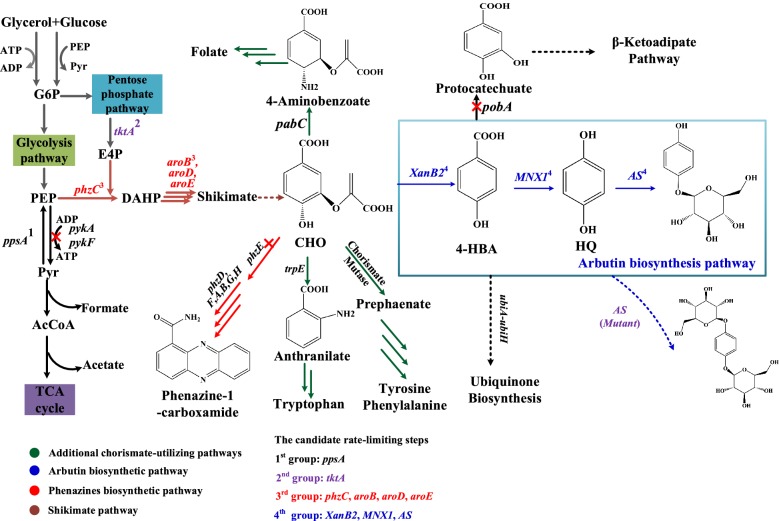
A schematic representation of modular engineering approaches for arbutin biosynthesis in *P. chlororaphis* P3. G6P, glucose 6-phosphate; PEP, phosphoenolpyruvate; E4P, erythrose 4-phosphate; DAHP, 3-deoxy-d-arabino-heptulosonate-7-phosphate; CHO: chorismate; 4-HBA: 4-hydroxybenzoate acid; HQ: hydroquinone. *pykA pykF*, pyruvate kinase; *tktA*, pyruvate synthase; *phzC*, 2-keto-3-deoxy-d-arabino-heptulosonate-7-phosphate synthase; *aroB*: 3-dehydroquinate synthase; *aroD*: 3-dehydroquinate dehydratase; *aroE*: dehydroshikimate reductase; *phzE*, anthranilate synthase; *pobA*: 4-hydroxybenzoate 3-monooxygenase; *trpE*: anthranilate synthase; *XanB2*, chorismate-pyruvate lyase; *MNX1*, 4-hydroxybenzoate 1-hydroxylase; *AS*, glucosyltransferase

In this context, the development of a plasmid-independent strategy for optimized gene expression appears as an efficient strategy to meet the accelerating demand for the green industry. Further, the direct integration of relevant genes into the host chromosome displays additional advantages in terms of stability and releases metabolic burden over the use of vectors [16]. Herein, *P*. *chlororaphis* P3 was metabolically engineered for arbutin biosynthesis by a plasmid-free strategy (Fig. 1). The genes for arbutin biosynthesis were expressed in phenazine synthesis gene cluster under the native promoter *P*_*phz*_. The engineered *P. chlororaphis* P3-Ar5 strain resulted in 6.79 g/L arbutin production with the productivity of 0.094 g/L/h from glucose and 4-HBA mixed fed-batch fermentation. The high arbutin titer achieved in our plasmid-free engineered strain demonstrates the feasibility of the large-scale microbial biosynthesis of arbutin from cheaper and sustainable carbon sources.

## Results

### Creation of a synthetic pathway for arbutin production from 4-HBA

**Fig. 2:**
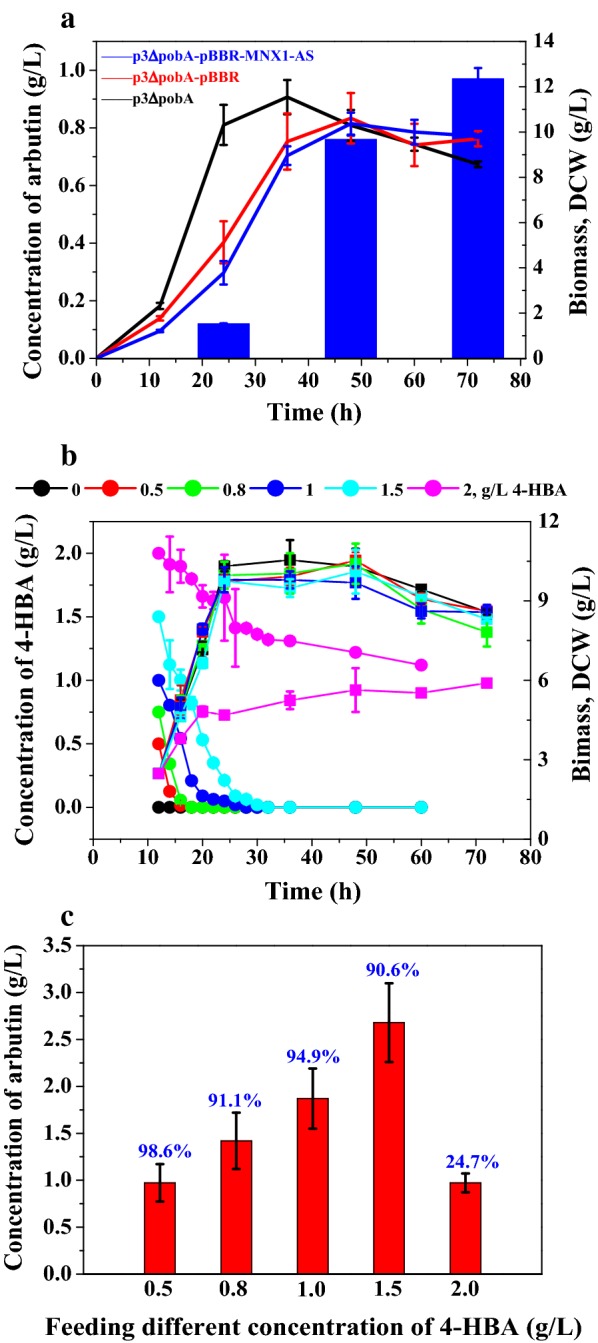
Arbutin production from 4-HBA. **a** Growth profile of different engineered strains, DCW (line), concentration of arbutin (column); **b** concentration of 4-HBA checked when feeding different concentration of 4-HBA to the culture (circular), growth profile of P3-Ar0, DCW (square); **c** arbutin conversion from 4-HBA measured at 48 h

Generally, two enzymatic steps including decarboxylation of 4-HBA to quinol by *MNX1* and glycosylation of quinol into arbutin with *AS* are involved in constructing an arbutin biosynthetic strain from 4-HBA. It is reported that, both *MNX1* and *AS* were efficient enough for arbutin synthesis from 4-HBA when expressed in *E*. *coli* [11]. To construct a *P*. *chlororaphis* strain capable of producing arbutin from 4-HBA, *MNX1* and *AS* were cloned into pBBR1MCS. The parent strain P3Δ*pobA* with a defect in the metabolism of 4-HBA was used in this study. Thus, we transformed pBBR-MNX1–AS into *P*. *chlororaphis* P3Δ*pobA* to construct P3Δ*pobA*-pBBR-MNX1–AS. Figure 2a displayed the cell growth and arbutin production when cultivation of P3Δ*pobA*-pBBR-MNX1–AS in KB medium supplemented with 0.5 g/L 4-HBA for 72 h. Evidently, the cell growth was impaired when introducing exogenous plasmid, and the arbutin was successfully produced but at a later stage of fermentation than that to other products reported in our earlier study [15, 17, 18] in the presence of inducer and antibiotics. Chromosomal integration is advocated as the preferred strategy to overcome this drawback. As demonstrated previously, phenazine biosynthesis genes are expressed under the strong phenazine synthesis promoter (*P*_*phz*_) and the native promoter is a powerful tool for the construction of new pathway [17]. On this basis, the phenazine biosynthetic genes are driven by stronger promoter that could be candidates for substitution with target genes under the control of native strong promoter *P*_*phz*_. At the first step, *MNX1* and *AS* were integrated into *phzA* and *phzB* locus individually. Moreover, a plasmid-free derivative strain P3-Ar0 was constructed by the inactivation of *pobA* encoding 4-hydroxybenzoate 3-monooxygenase. In order to corroborate the efficiency of these two enzymes which were expressed under native promoter *P*_*phz*_ in *P*. *chlororaphis*, different concentrations of 4-HBA (ranging from 0 to 2 g/L) were supplemented to the medium after culturing P3-Ar0 for 12 h. Notably, 4-HBA (0–1.5 g/L) was almost completely converted to arbutin with a conversion rate of more than 90% after 60 h (Fig. 2b, c), nevertheless, higher concentration of 4-HBA (2 g/L) inhibited the growth of *P*. *chlororaphis* and thus limited the conversion rate for arbutin to 24.7%. The culture supernatant of P3-Ar0 was analyzed by UPLC/MS to confirm the arbutin production. Specific peak of arbutin was observed at ~ 5.6 min with the m/z = 317.08 (negative ions, Additional file 1 Figure S1). These results suggested that *MNX1* and *AS* were efficient enzymes for arbutin biosynthesis when expressed on phenazine biosynthetic locus from 4-HBA under the native promoter *P*_*phz*_.

### Designing a biosynthetic pathway for arbutin production from glycerol

**Fig. 3:**
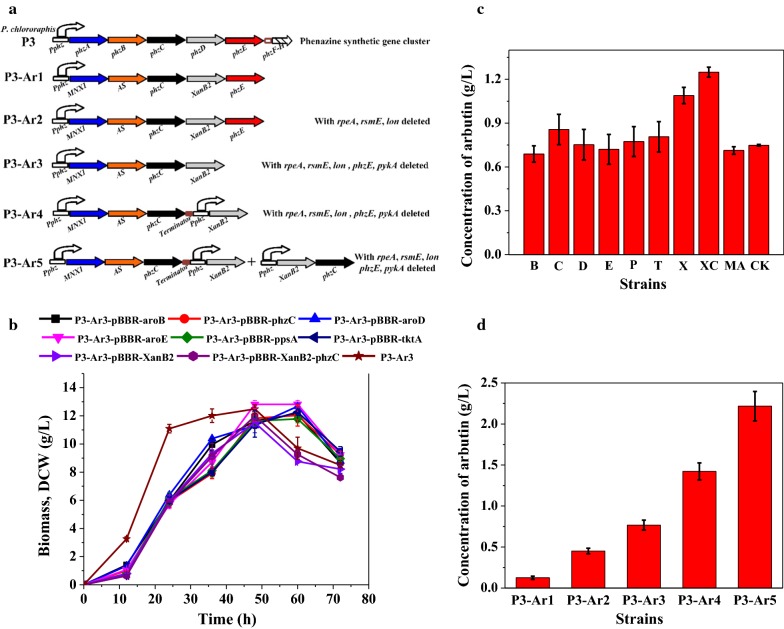
A summary of the steps in the genetic and metabolic engineering of *P*. *chlororaphis* for arbutin production. **a** The construction step for enhanced arbutin production; **b** growth profile of the arbutin-producing transformants harboring plasmid; **c** the amount of arbutin produced by transformants harboring plasmid checked at 48 h. B, P3-Ar3-pBBR-*aroB*; C, P3-Ar3-pBBR-*phzC*; D, P3-Ar3-pBBR-*aroD*; E, P3-Ar3-pBBR-*aroE*; P, P3-Ar3-pBBR-*ppsA*; T, P3-Ar3-pBBR-*tktA*; X, P3-Ar3-pBBR-XanB2; XC, P3-Ar3-pBBR-XanB2–*phzC*; MA, P3-Ar3-pBBR-MNX1–AS; CK, P3-Ar3.; **d** the yield of arbutin produced by engineered *P*. *chlororaphis* at 48 h. Data are presented as the mean ± standard deviation of three independent experiments (n = 3)

When *MNX1* and *AS* were co-expressed in *P. chlororaphis* under the control of *P*_*phz*_, the conversion yield increased to more than 90% for arbutin biosynthesis from 4-HBA. Then, we focused on constructing an arbutin biosynthetic pathway from glycerol via the shikimate pathway. In our earlier study, several chorismatases were expressed in *P. chlororaphis* on the different chromosomal locus, when *XanB2* from *X. oryzae* pv. *oryzae* was expressed on *phzA* locus under the control of *P*_*phz*_, more than 1.5 g/L 4-HBA was produced using glycerol as a carbon source [17]. XanB2 is an ideal enzyme for designing arbutin biosynthesis pathway, and therefore it was employed to generate 4-HBA for arbutin synthesis. Then the heterogeneous *XanB2* was genetically introduced into strain P3-Ar0 to result derivative strain P3-Ar1 by replacing *phzD*, in order to improve the 4-HBA production for arbutin synthesis. 3-deoxy-d-arabino-heptulosonate-7-phosphate (DAHP) synthase encoded by *phzC* is the first enzyme that catalyzes the fisrt step of the shikimate pathway. It is fully essential for the chorismate-derived products synthesis which located in phenazine synthetic gene cluster in *P*. *chlororaphis* (Fig. 3a). Thus, arbutin synthetic genes including *MNX1*, *AS*, *XanB2* and endogenous *phzC* were expressed under the control of native strong promoter *P*_*phz*_ showing in Fig. 3a. Afterwards the strain P3-Ar1 was cultivated in KB medium to evaluate the arbutin production capacity from glycerol. After chemostat culture for 72 h, 0.126 g/L arbutin was achieved with the yield of 0.007 g/g glycerol and productivity of 0.0018 g/L/h. On the basis of the results, a plasmid-free biosynthetic pathway for arbutin production was well constructed in *P*. *chlororaphis* via the high-efficient shikimate pathway using a native promoter *P*_*phz*_. The low titer may be attributed to the inadequate supply of key precursor 4-HBA.

### Disruption of negative regulatory genes and competitive pathway to enhance arbutin production

The biosynthetic pathway for arbutin production was successfully constructed with *MNX1*, *AS XanB2* and endogenous *phzC* expressed on the chromosome under the control of *P*_*phz*_. To further enhance the arbutin production, more strategies were employed in this study as follows. In our previous study, the production of 2-hydroxyphenazine increased from 0.035 to 0.301 g/L following the deletion of negative regulators involved in the regulation of secondary metabolism, RpeA (encoded by *rpeA*) and RsmE. And one ATP-dependent protease Lon encoded by *lon* that stimulates production of secondary metabolites by inhibiting the Gac/Rsm signal transduction system as reported *P*. *chlororaphis* was also deleted to enhance the 2-hydroxybenazine production [18]. On this basis, *lon*, *rpeA*, and *rsmE* were deleted sequentially in P3-Ar1 to construct P3-Ar2. To weaken the competitive pathway of arbutin synthesis, *pykA* encoding pyruvate kinase which converts metabolic precursor phosphoenolpyruvate (PEP) to the tricarboxylic acid (TCA) cycle, and *phzE*, involved in converting chorismate for phenazine biosynthesis, were deleted for enhancing precursor PEP and chorismate supply in P3-Ar2 to yield P3-Ar3.

Fermentation experiments revealed that after 72 h of cultivation in KB medium, the highest titer of arbutin was recorded to be 0.45 g/L and 0.77 g/L for P3-Ar2 and P3-Ar3, respectively (Fig. 3). At this stage, P3-Ar3 could produce 0.77 g/L arbutin, a 5.95-fold increase from the initial arbutin-producing strain, with the yield of 0.043 g/g glycerol, and productivity of 0.011 g/L/h. Notably, arbutin was the first reported glycoside produced in *Pseudomonas* strain independent of plasmid and inducer.

### Improvement of a rate-limiting step in arbutin production

To further enhance the arbutin production, we attempted to identify and improve the rate-limiting step in the synthetic pathway of arbutin production. On the basis of previous study for enhanced shikimate pathway [17–19], four categories of genes were selected from the gluconeogenesis pathway, pentose phosphate pathway, shikimate pathway and “chorismate-arbutin” pathway. The first group, PEP synthase encoded by *ppsA* was generally used to improve the precursor PEP. The second group, transketolase (TktA) was selected as a candidate to enhance the E4P availability. The third group including genes *phzC*, *aroB*, *aroD*, *aroE* were chosen to strengthen the shikimate pathway [18]. The fourth group included chorismate pyruvate lyase *XanB2* which was chosen to improve 4-HBA production, *MNX1* and *AS* were chosen for enhancement the conversion of 4-HBA to arbutin. All these genes were individually cloned into pBBR1MCS and introduced into P3-Ar3 by electrotransformation. Figure 3b clearly depicts that the growth of cells was delayed when introduced pBBR1MCS, which was in accordance with our earlier study [17]. To evaluate the effects of these candidates, each of the transformants was cultured in KB medium, and the production of arbutin was detected by HPLC. It can be seen in Fig. 3c that no significant increase in arbutin production was observed by the overexpression of *aroB*, *aroD*, *aroE*, *ppsA*, *tktA*, *MNX1* and *AS*. The strain P3-Ar3-pBBR-*phzC* produced 0.86 g/L arbutin, which was 14.5% higher than that of P3-Ar3. The final concentration of arbutin produced by strain P3-Ar3-pBBR-*XanB2* reached 1.09 g/L. The best arbutin producing derivative P3-Ar3-pBBR-*XanB2*–*phzC*, accumulated up to 1.25 g/L arbutin when *XanB2* and *phzC* were co-expressed, which was 67% higher than that of P3-Ar3. On the basis of these experimental results, *XanB2* and *phzC* were selected for overexpression on the chromosome as reported previously [17], *phzC* was overexpressed for the increase of precursor DAHP, and the overexpression of *XanB2* will improve 4-HBA production.

When feeding different concentration of 4-HBA to P3-Ar0 culture, most of 4-HBA was converted to arbutin without 4-HBA (or HQ) accumulation indicating that “4-HBA-Arbutin” is not a rate-limiting step in the whole biosynthetic pathway. The higher efficiency of MNX1 and AS could provide a strong driving force for arbutin biosynthesis from 4-HBA. Towards a strong expression of the heterologous *XanB2* gene, *XanB2* and *P*_*phz*_ promoter were integrated into *phzD* locus yielding strain P3-Ar4 (Fig. 3a). When P3-Ar4 was fermented in KB medium, 1.42 g/L arbutin was produced with a yield of 0.079 g/g glycerol and the titer improvement was 11.3-fold in contrast to the original strain P3-Ar1. It was in agreement with previous observations that increasing the expression of *XanB2* would enhance the production of 4-HBA derivatives [17]. Furthermore, *XanB2* and *phzC* were cloned into *pykA* locus under the control of native promoter *P*_*phz*_, and the derivative strain P3-Ar5 was obtained. In consequence, the highest titer of the recombinant strain P3-Ar5 was 2.22 g/L after culturing, with the yield of 0.12 g/g glycerol and productivity of 0.031 g/L/h (Fig. 3d). The step-wise approach resulted in a 17.1-fold improvement for arbutin production compared to the parent strain.

### Cultivation optimization for arbutin production

**Fig. 4:**
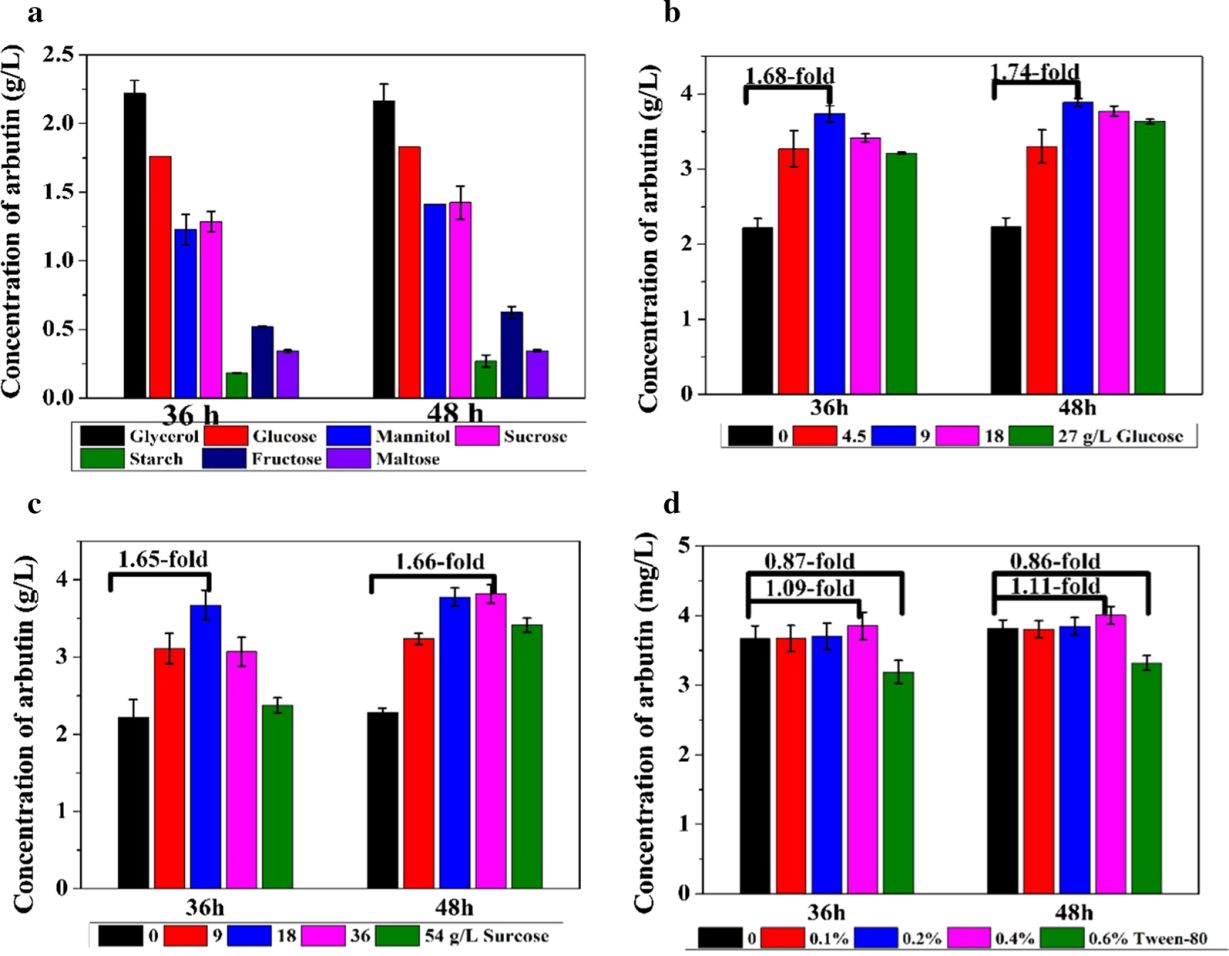
Culture profile of P3-Ar5 under different carbon source conditions. **a** Fermentations were carried out in KB medium using different carbon sources to replace glycerol; **b** fermentations were carried out in KB medium supplemented with different concentration of glucose; **c** fermentations were carried out in KB medium supplemented with sucrose medium; **d** fermentations were carried out in KB medium supplemented with glucose and Tween-80. Data are presented as the mean ± standard deviation of three independent experiments (n = 3)

It is possible that 4-HBA and glycosylation donor of arbutin were synthesized from glycerol when the whole pathway was constructed, the needed glycosylation donor was produced with the cost of a decrease in the carbon flow from a carbon source to 4-HBA. To enhance the carbon flow for arbutin synthesis, different carbon sources including glucose (18 g/L), fructose (18 g/L), sucrose (36 g/L), maltose (36 g/L), starch (36 g/L) and mannitol (18 g/L) were tested for arbutin production. In consonance with previous reports [17], glycerol was found to be an ideal carbon source for chorismate derivatives production based on the shikimate pathway in *P*. *chlororaphis* (Fig. 4a). The arbutin titer was quite lower in fructose or maltose-based medium in contrast to glucose as a sole carbon source, indicating that *α*-sugar is used for arbutin biosynthesis rather than *β*-sugar. Therefore, glucose was selected as a glycosylation donor to increase the production of arbutin. For this, different concentrations of glucose were incorporated to KB medium (i.e. 4.5 g/L, 9 g/L, 18 g/L, and 27 g/L), and the concentration of arbutin was analyzed. Remarkably, the arbutin production increased to the highest titer 3.89 g/L by adding 9 g/L glucose (Fig. 4b). Considering the efficiency of glucose and carbon source preference, it is found that glucose serves as not only carbon source for cell growth but also the substrate for arbutin biosynthesis. Another carbon source, sucrose as a disaccharide was also tested in the same manner by adding to KB medium with different concentrations (9 g/L, 18 g/L, 36 g/L, and 54 g/L sucrose), and results are shown in Fig. 4c. The highest titer of arbutin (3.81 g/L) was achieved by feeding 18 g/L sucrose and the result was similar to that feeding 9 g/L glucose. Additionally, when using 27 g/L glycerol or 27 g/L glucose as sole carbon source, the titer of arbutin was 2.52 g/L and 2.01 g/L with the productivity of 0.035 g/L/h and 0.028 g/L/h, respectively, which was significantly lower than that in KB medium with glucose.

The surfactant is capable of accelerating substances delivery in the fermentation process by improving the permeability of the cell membrane [20]. As reported, 0.2% (w/v) of Tween-80 promoted the arbutin production compared with cetyltrimethylammonium bromide (CTAB), sodium dodecyl sulfate (SDS), sophorose lipid and polyester 30 [21]. Supplementation of the surfactant Tween-80 is considered as a common strategy to increase the production of arbutin. Therefore, the effect of different concentration of Tween-80 was evaluated in the fermentation of P3-Ar5, and the arbutin production increased when feeding 0.4% Tween-80 (Fig. 4d). The highest production of arbutin was 4.10 g/L, with the productivity of 0.057 g/L/h, with a 9% improvement. However, an elevated concentration of surfactant beyond 0.6% decreased the yield by 13% (Fig. 4d), presumably due to the toxic effects of Tween-80 on *P*. *chlororaphis* cells.

**Fig. 5:**
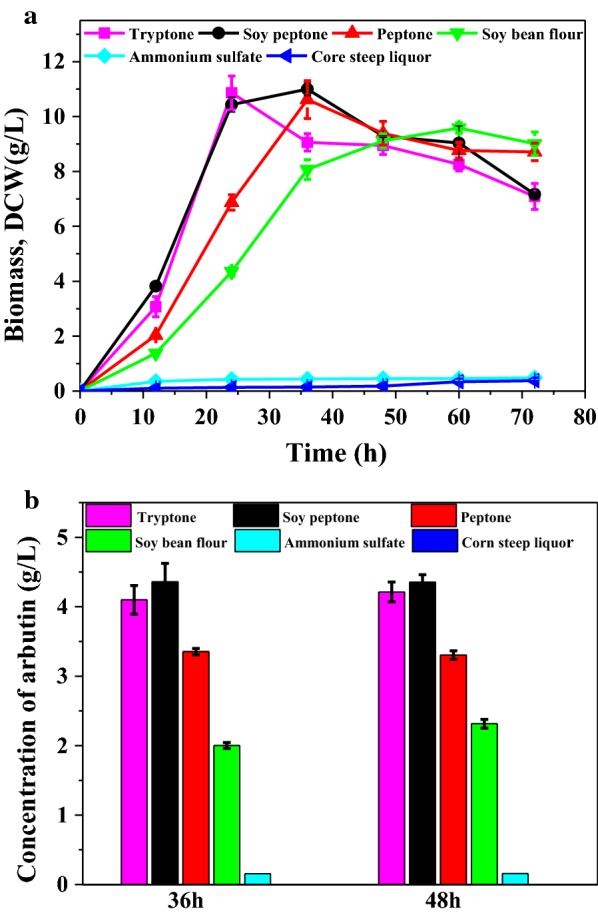
Culture profile of P3-Ar5 under different nitrogen source conditions in KB medium adding 9 g/L glucose and 0.4% Tween-80. **a** Growth curve of P3-Ar5 under different nitrogen source conditions; **b** Arbutin produced by P3-Ar5 under different nitrogen source conditions. Data are presented as the mean ± standard deviation of three independent experiments (n = 3)

To further improve the cost-effectiveness of arbutin production, different economic nitrogen sources including ammonium sulfate, corn steep liquor, peptone, soy peptone, and soybean flour, were tested in this study. Figure 5 represents that the arbutin production using soy peptone was comparable to that using tryptone. The soy peptone had no negative effect on the growth of engineered strain, and the titer of arbutin increased 10% to 4.36 g/L. In contrary to our expectations, *P*. *chlororaphis* could not metabolize corn steep liquor as a nitrogen source to grow. As a conclusion, the optimal medium for arbutin production was chosen as KBG medium (9 g/L glucose, 18 g/L glycerol, 20 g/L soy peptone, 0.4% Tween-80, 0.732 g/L MgSO_4_, 0.673 g/L K_2_HPO_4_·3H_2_O). Under the optimized medium in shake-flask cultivation of P3-Ar5, 4.36 g/L arbutin could be produced with the productivity of 0.061 g/L/h, the titer increased 34.7-fold in contrast with the initial strain and was comparable to the plasmid-based synthesis of arbutin in *E*. *coli* [11].

### Improving the arbutin yield by co-feeding 4-HBA and glucose

**Fig. 6:**
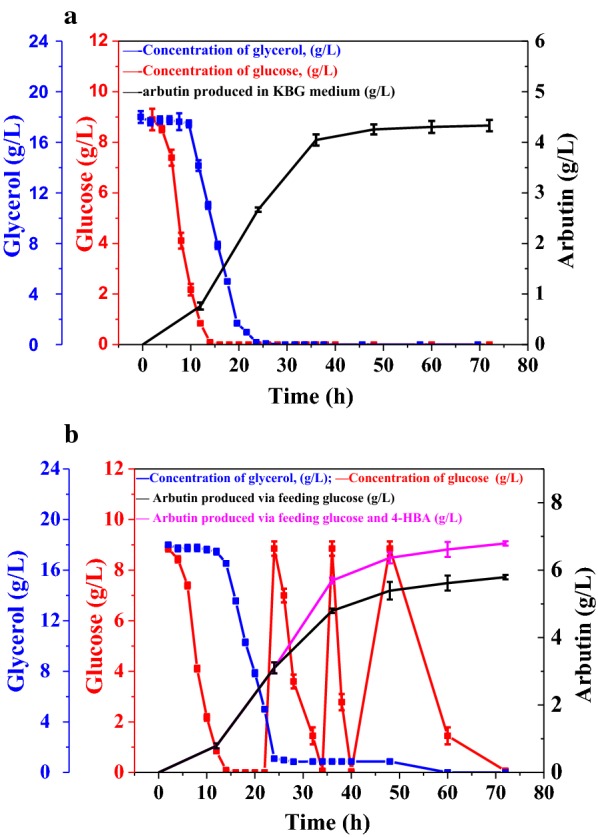
Culture profile of P3-Ar5. **a** Culturing P3-Ar5 at the initial glycerol concentration of 18 g/L and glucose 9 g/L; **b** culturing P3-Ar5 by feeding glucose and 4-HBA mixture at the final concentration of glucose 9 g/L, 4-HBA 0.8 g/L per 24 h. Data are presented as the mean ± standard deviation of three independent experiments (n = 3)

**Fig. 7:**
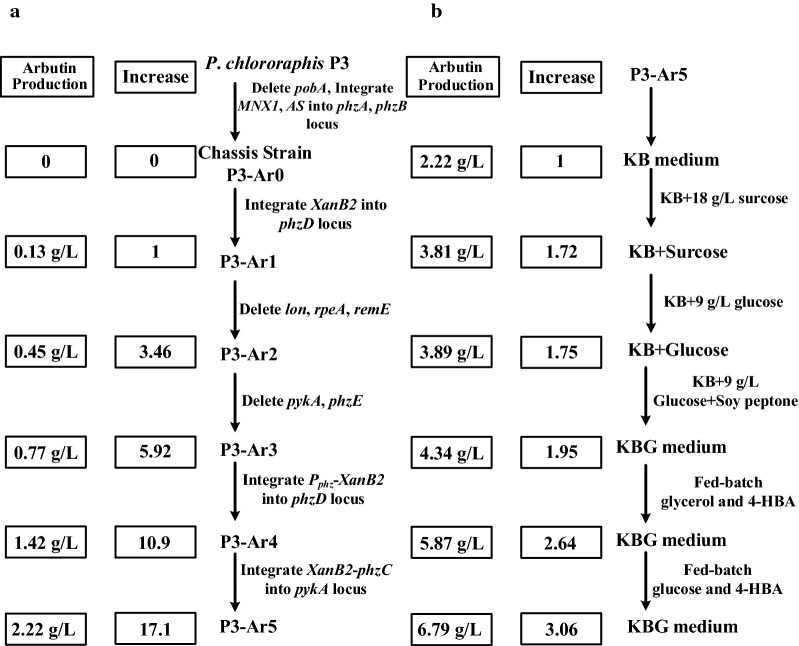
A summary of steps for improvement of the production of arbutin. **a** On the basis of genetic and metabolic engineering; **b** on the basis of medium factor optimization and fed-batch fermentation

To evolve into an economically feasible candidate for industrial arbutin production, P3-Ar5’s productivity needs to be maximized. It can be concluded from the checked concentration of glycerol and glucose (Fig. 6a), after 13 h culturing of P3-Ar5, the engineered strain would begin to metabolize glycerol following glucose depletion. No significant change in pH value of P3-Ar5 cultures was detected upon glucose consumption (Additional file 1: Figure S2) because more metabolic flux has been distributed into producing arbutin rather than generating acetate. The concentration of glucose can also be used to direct the glucose assimilation through the fermentation process. Different feeding strategies were analyzed to further improve the production of arbutin. Firstly, the mixture of glycerol (18 g/L) and glucose (9 g/L) was supplemented to the culture every 24 h. The data showed that the production of arbutin increased with feeding carbon source, the titer was 5.82 g/L with the productivity of 0.097 g/L/h at 60 h, which represented a 34% improvement in comparison with the batch culture. Secondly, the precursor 4-HBA and glycerol were supplemented to the culture. When feeding different concentration of 4-HBA to the culture, it can be seen that when adding 0.8 g/L 4-HBA after pre-cultivation of 12 h, 4-HBA could be metabolized within 4 h (Fig. 2b), however, there was some 4-HBA left in the supernatant when adding more than 1 g/L 4-HBA after culturing 12 h. Therefore, glycerol, or glycerol and 4-HBA mixture were co-supplemented to the culture at the final concentration of 18 g/L glycerol or glycerol (18 g/L) and 4-HBA (0.8 g/L) after cultivating P3-Ar5 for 24 h. The titer of arbutin was improved to 5.02 g/L, with the productivity of 0.084 g/L/h, when glycerol was added to the medium at a final concentration of 18 g/L every 24 h. However, when co-feeding 4-HBA and glycerol every 24 h, 5.87 g/L arbutin was produced with the productivity of 0.098 g/L/h after fermentation. Last but not the least, glucose, or glucose and 4-HBA mixture were co-supplemented to the culture at a final concentration of 9 g/L glucose, and 0.8 g/L 4-HBA after cultivating P3-Ar5 for 24 h. As can be seen in Fig. 6b, the arbutin titer was significantly improved (5.62 g/L). When feeding 4-HBA and glucose at 24 h, after 12 h culturing most of the glucose and 4-HBA were consumed with 5.69 g/L arbutin production at 36 h with the productivity of 0.16 g/L/h, and the glucose uptake rate was recorded to be 383.3 mg/L/h. When feeding at 36 h, 6.34 g/L arbutin was achieved with the productivity of 0.13 g/L/h at 48 h with the glucose uptake rate 410.9 mg/L/h. The feeding at 48 h yielded 6.61 g/L arbutin with the productivity of 0.11 g/L/h at 60 h. Finally, 6.79 g/L arbutin was produced with the productivity of 0.094 g/L/h. Interestingly, when 4-HBA was supplemented to the medium, the pH of fermentation supernatant decreased but restored to normal (pH = 6.8) after complete 4-HBA consumption. This phenomenon will contribute for arbutin production via monitoring pH to guide 4-HBA fed-batch in fermentation industry. The production of arbutin increases by feeding 4-HBA, and 0.8 g/L 4-HBA can be efficiently catalyzed by *MNX1* within 12 h. These results revealed that the co-expression of *XanB2*, *MNX1,* and *AS* would be quite efficient for arbutin production using chassis strain *P*. *chlororaphis* P3. The production of arbutin was significantly enhanced when co-feeding glucose and 4-HBA in comparison with the glycerol and glucose or glycerol and 4-HBA mixed feeding (Fig. 7). One possible explanation is that glucose was the preferred carbon source of P3-Ar5; the glycerol was utilized after glucose depletion, and produced twice as much reducing equivalent as glucose when converted to PEP. Thereby, co-feeding glucose and 4-HBA was more useful for providing the key precursors for arbutin biosynthesis.

Finally, P3-Ar5 was tested for the stability of the integrated genes. Results suggested that arbutin productivity was consistent after culturing the engineered derivative strain for many generations (Additional file 1: Figure S3). Further, the tolerance of P3-Ar5 to arbutin was also tested. Based on the results, no significant changes were detected and no noticeable arbutin degradation was observed in the production conditions (Table 1). The current study proposed a stable and enhanced arbutin biosynthesis strategy, which displays a high biotechnological perspective.

**Table 1:** Growth kinetic parameters of P3-Ar5 feeding different concentration of arbutin

P3-Ar5	Concentration of arbutin feeding to the culture (g/L)				
	2	3	4	5	6
Specific growth rate (h^−1^)	0.133 ± 0.002	0.132 ± 0.002	0.133 ± 0.003	0.131 ± 0.002	0.132 ± 0.001
Arbutin checked^a^ (g/L)	6.26 ± 0.066	7.66 ± 0.046	8.35 ± 0.065	9.34 ± 0.032	9.99 ± 0.075

Data are presented as the mean ± standard deviation of three independent experiments (n = 3)

^a^Arbutin produced after 60 h culturing supplemented with different concentration of arbutin to KBG medium

## Discussion

In the previous study, a variety of proteins and genes related to the enhancement of chorismate derivative (PCN) production have been identified in *P*. *chlororaphis* P3 [15], which makes the strain *P*. *chlororaphis* P3 feasible to be used as a potential candidate for genetic modification to obtain a high-yielding mutant exploiting the high-efficient shikimate pathway. The efficiencies of *MNX1* and *AS* have been well evaluated by plasmid-based expression in *E*. *coli* for the biosynthesis of arbutin [11], and *XanB2* was found to be one of the best chorismatase for 4-HBA production in *P*. *chlororaphis* in our earlier study [17]. To address the increasing green industry’s demand and reduce the use of antibiotics in the fermentation bioprocesses, the plasmid-free genes integration is a commendable approach for high-level production of target compounds [16, 22]. In this work, arbutin was synthesized without the use of plasmid and inducer via expressing exogenous *MNX1*, *AS* and *XanB2* on the chromosome of *P*. *chlororaphis* P3. To the best of our knowledge, glycosides were first time produced in *P*. *chlororaphis* under the native promoter independent of plasmid and inducer. Actually, *MNX1* and *AS* could be quite efficient when expressed in the chromosome of *P*. *chlororaphis* comparing with plasmid-based expression by feeding 4-HBA experiments (Fig. 2b, c). Meanwhile, chromosomal integration is preferred to integrative plasmid-based expression as reported for arbutin synthesis [11].

Numerous studies reporting deregulation of the feedback inhibition, enhancement of the shikimate pathway, and increased accumulation of precursors PEP and E4P have been attempted to increase the production of target biologicals of shikimate pathway [23–26]. As competitive branches, the disruption of aromatic amino acid (Tyr, Trp, and Phe) biosynthesis pathway might contribute to the improvement of chorismate derivatives [27, 28]. A *phzE* gene inactivated mutant (HT66Δ*phzE*) was used to assess the competitive pathway of chorismate in the previous study, however, no significant accumulation of aromatic amino acids was detected [17]. In this study, it is not necessary to knock out the aromatic amino acid pathway avoiding auxotrophic strain. Three negative regulatory genes *lon*, *rpeA* and *rsmE* were deleted individually as previously reported [18] to improve the production of arbutin with the 3.59-fold increase, though the improvement was lower than the 2-hydroxyphenazine production. We speculated that these regulators can regulate the expression of arbutin biosynthetic gene operator, but underlying mechanism needs to be excavated further. By fine-tuning of biosynthesis pathway, the limiting rate was enhanced by multiple chromosomal integrations of *XanB2* and *phzC* under the strong promoter *P*_*phz*_ to increase 22.43-fold arbutin production. Considering that glucose and 4-HBA serving as substrates for arbutin biosynthesis, optimization of the concentration of glucose at 9 g/L further improved the production of arbutin (Fig. 4). The effect was similar when feeding 18 g/L sucrose, because AS catalyzes specifically to form α–arbutin, and we found that α-sugar was used for arbutin biosynthesis rather than *β*-sugar, which is strongly supported by our observation as shown in Fig. 4. These results are consistent with the earlier report that increasing the glucose concentration could improve arbutin production in *E*. *coli* [11]. It is noteworthy that supplement of glucose and 4-HBA mixture in the fermentation process will further increase the yield of arbutin in comparison with feeding glucose and glycerol or glycerol and 4-HBA. A high titer of 6.79 g/L arbutin was achieved with the productivity of 0.094 g/L/h, when co-feeding 4-HBA and glucose at 24 h, 36 h, and 48 h. This titer of arbutin production based on plasmid-free strategy is the highest in *Pseudomonas* reported up to date [11]. Moreover, arbutin glycoside was first reported to be produced by *P*. *chlororaphis* independent of plasmid and inducer.

In this study, arbutin was synthesized using the promising intermediate 4-HBA as a precursor, which has been developed for several value-added bioproducts [14]. As reported previously, several 4-HBA production systems have been constructed. The recombinant *Burkholderia glumae* could accumulate 2.73 g/L 4-HBA from *p*-coumaric acid [29], the recombinant *P*. *putida* could produce 1.8 g/L 4-HBA from glycerol [30], and the engineered *E*. *coli* could produce 12 g/L 4-HBA in fermenter [31]. As a vital intermediate platform chemical, 4-HBA could be easily utilized and converted into more valuable biological products [14].

Further metabolic evolution and engineering are expected to improve co-utilizing of glycerol and glucose in *P*. *chlororaphis*. Since glycerol has a higher degree of reduction than glucose or xylose for the production of reduced biologicals [32], it seems a straightforward carbon source for the production of redox-demanding compounds through fermentation processes [33]. It is useful for the enhanced production of chemicals from glycerol via improving the glycerol metabolic capability. Therefore, co-utilization of glycerol and glucose should be studied to enhance the production of arbutin. *P*. *chlororaphis* shows the potential ability for aromatic compounds degradation such as 4-HBA, 3-hydroxybenzoic acid (3-HBA), 3,4-dihydroxybenzoic acid (PCA), catechol (MC) and gallic acid [17]. It is of great significance to combine a secondary metabolite pathway and the benzene ring cleavage pathway for biosynthesis of various value-added chemicals, such as muconic acid and maleate [17, 26].

Directed evolution is a widely-used engineering strategy for enhancement of the stabilities or biochemical functions of proteins [34]. In the arbutin biosynthesis pathway, *AS* encoding glucosyltransferase displayed a higher specificity towards HQ and low activity towards other HQ analogs capable of accepting uridine diphosphate glucose (UDPG) from glucose as glycosyl donor to generate arbutin [35]. Through the directed evolution of *AS*, HQ may accept two UDPG and yield a series of new uncharacterized 4-HBA derivatives (Fig. 1).

## Conclusions

In summary, a novel plasmid-free biosynthetic pathway was constructed for the stable and high-level biosynthesis of arbutin in *P*. *chlororaphis*. Instead of being plasmid and inducer dependent, the metabolic engineering approach and medium factor optimization used to fine-tune the biosynthetic pathway markedly enhanced the arbutin production. The engineered *P. chlororaphis* P3-Ar5 strain could accumulate up to 6.79 g/L arbutin with a 54-fold increase in arbutin production over the original strain based on glucose and 4-hydroxybenzoic acid mixed fed-batch fermentation. This work is useful to strengthen the microbial secondary metabolism for high-level production of plant-derived and natural products in environmental microorganisms. We believe that the high titer achieved in this plasmid-free pathway offers a great opportunity for industrial scale production of arbutin and other value-added compounds.

## Methods

### Strains, plasmids, medium and culture conditions

All the bacterial strains used and engineered in this study are listed in Table 2. The oligonucleotides are summarized in Additional file 1: Table S1. Luria–Bertani (LB) medium (Tryptone 10 g, Yeast extract 5 g, NaCl 10 g, g/L) was used for the culture of *E. coli* (37 °C) and *P. chlororaphis* (28 °C) during the construction of mutants. King’s medium B (KB) medium (Glycerol 18 g, Tryptone 20 g, MgSO_4_·7H_2_O 1.498 g, K_2_HPO_4_ 0.514 g, g/L) and KBG (Glycerol 15 mL, Soy peptone 20 g/L, Glucose 9 g/L, 0.4% Tween-80, MgSO_4_·7H_2_O 1.498 g/L, K_2_HPO_4_ 0.514 g/L) were used as a fermentation medium for the production of arbutin in shake flask (250 mL) by *P. chlororaphis*. Where applicable, 100 mg/L ampicillin or 50 mg/L kanamycin was added to the medium for selection. For shake-flask fermentation, *P*. *chlororaphis* and its derivatives were activated at 28 °C overnight in agar media. A single colony from Petriplates was inoculated in 50 mL flasks for 12 h at 28 °C with 200 rpm of shaking. Portions of these cultures were then inoculated into 250 mL baffled erlenmeyer flasks containing 60 mL specific medium to achieve an initial OD_600_ of 0.02 [17]. The fermentation process was then initiated and samples were collected every 12 h for the determination of cell growth and arbutin concentration. Dry cell weight (DCW) in KB medium was calculated from the optical density at 600 nm (1OD_600_ = 0.4135 g DCW/L). When genes were expressed in pBBR1MCS vector, isopropyl *β*-d-1-thiogalactopyranoside (IPTG) was added to the culture at a final concentration of 0.1 mM after 6 h of cultivation.

**Table 2:** Main strains and plasmids used and developed in this study

	Description	Source
Strains		
S17-1 (λ pir)	*E*. *coli* res^−^ pro mod^+^ integrated copy of RP4, mob^+^, used for incorporating constructs into *P*. *chlororaphis*	Lab stock
*P. chlororaphis* HT66	*P. chlororaphis* wild-type, PCN, Ap^r^, Sp^r^	Lab stock
*P. chlororaphis* P3	A mutant from *P*. *chlororaphis* HT66 with a high PCN production	Lab stock
HT66-4X_PD_	*P. chlororaphis* HT66, *Pphz*–*XanB2* co-expressed to insert *phzD* locus	Lab stock
HT66Δ*phzE*	*P. chlororaphis* HT66 with *phzE* deleted	This study
P3Δ*phzE*	*P. chlororaphis* P3 with *phzE* deleted	This study
P3Δ*pobA*	*P. chlororaphis* P3 with *pobA* deleted	This study
P3-Ar0	P3ΔpobA, *MNX1* inserted to *phzA* locus, *AS* inserted to *phzB* locus	This study
P3-Ar1	P3-Ar0, *XanB2* inserted to *phzD* locus, arbutin synthetic genes including *MNX1*, *AS*, *phzC* and *XanB2* were expressed using *P*_*phz*_	This study
P3-Ar2	P3-Ar1, with *rpeA*, *lon* and *remE* deleted	This study
P3-Ar3	P3-Ar2, with *pykA*, *phzE* deleted	This study
P3-Ar3-pBBR-*aroB*	P3-Ar3, harboring plasmid pBBR-*aroB*	This study
P3-Ar3-pBBR-*aroD*	P3-Ar3, harboring plasmid pBBR-*aroD*	This study
P3-Ar3-pBBR-*phzC*	P3-Ar3, harboring plasmid pBBR-*phzC*	This study
P3-Ar3-pBBR-*aroD*	P3-Ar3, harboring plasmid pBBR-*aroD*	This study
P3-Ar3-pBBR-*aroE*	P3-Ar3, harboring plasmid pBBR-*aroE*	This study
P3-Ar3-pBBR-*ppsA*	P3-Ar3, harboring plasmid pBBR-*ppsA*	This study
P3-Ar3-pBBR-*tktA*	P3-Ar3, harboring plasmid pBBR-*tktA*	This study
P3-Ar3-pBBR-*XanB2*	P3-Ar3, harboring plasmid pBBR-*XanB2*	This study
P3-Ar3-pBBR-*XanB2*–*phzC*	P3-Ar3, harboring plasmid pBBR-*XanB2*–*phzC*	This study
P3-Ar3-pBBR-*MNX1*–*AS*	P3-Ar3, harboring plasmid pBBR-*MNX1*–*AS*	This study
P3-Ar4	P3-Ar3, *P*_*phz*_–*XanB2* co-expressed to insert *phzD* locus	This study
P3-Ar5	P3-Ar4, *P*_*phz*_–*XanB2*–*phzC* co-expressed to insert *pykA* locus	This study
Plasmids		
pk18mobsacB	Broad-host-range gene replacement vector, Km^r^	Lab stock
pk18-*pobA*	pk18mobsacB containing *pobA* upstream and downstream, Km^r^	This study
pk18-*lon1*	pk18mobsacB containing *lon1* upstream and downstream, Km^r^	This study
pk18-*lon2*	pk18mobsacB containing *lon2* upstream and downstream, Km^r^	This study
pk18-*rpeA*	pk18mobsacB containing *rpeA* upstream and downstream, Km^r^	This study
pk18-*remE*	pk18mobsacB containing *remE* upstream and downstream, Km^r^	This study
pk18-*pykA*	pk18mobsacB containing *pykA* upstream and downstream, Km^r^	This study
pk18-*phzE*	pk18mobsacB containing *phzE* upstream and downstream, Km^r^	This study
pk18-*XanB2*	pk18mobsacB containing *XanB2*, *phzD* upstream and downstream, Km^r^	This study
pk18-*MNX1*–*AS*	pk18mobsacB containing *MNX1*–*AS*, *phzA* upstream and *phzB* downstream, Km^r^	This study
pk18-P_phz_–*XanB2*–*phzC*	pk18mobsacB containing *P*_*phz*_–*XanB2*–*phzC*, *pykA* upstream and downstream, Km^r^	This study
pBBR1MCS	T7 expression vector, Km^r^	Lab stock
pBBR-*aroB*	pBBR1MCS containing *aroB* for overexpression, Km^r^	This study
pBBR-*phzC*	pBBR1MCS containing *phzC* for overexpression, Km^r^	This study
pBBR-*aroD*	pBBR1MCS containing *aroD* for overexpression, Km^r^	This study
pBBR-*aroE*	pBBR1MCS containing *aroE* for overexpression, Km^r^	This study
pBBR-*ppsA*	pBBR1MCS containing *ppsA* for overexpression, Km^r^	This study
pBBR-*tktA*	pBBR1MCS containing *tktA* for overexpression, Km^r^	This study
pBBR-*XanB2*	pBBR1MCS containing *XanB2* for overexpression, Km^r^	This study
pBBR-*XanB2*–*phzC*	pBBR1MCS containing *XanB2* and *phzC* for overexpression, Km^r^	This study
pBBR-*MNX1*–*AS*	pBBR1MCS containing *MNX1* and *AS* for overexpression, Km^r^	This study

### Pathway and plasmid construction

All genes were PCR amplified with PrimerSTAR Max DNA Polymerase (TaKaRa Bio). Each gene was assembled with the respective plasmid using In-Fusion Cloning Kit (TaKaRa Bio). Chromosomal in-frame deletions of *pobA* were individually carried out as reported earlier [18]. *MNX1* from *Candida parapsilosis* CBS604 and *AS* from *Rauvolfia serpentine* were codon optimized and synthesized by Genewiz (Suzhou, China). To substitute gene *phzA* and *phzB* with *MNX1* and *AS,* a modified gene deletion version was used to amplify a 400–500 bp DNA fragment of *phzA* upstream, the open reading frame (ORF) of *MNX1*, *AS* and 400–500 bp DNA fragment of *phzB* downstream, above genes, were cloned into pk18mobsacB using In-Fusion Cloning Kit (TaKaRa Bio).

Other gene deletion or substitution derivatives were constructed in the corresponding strains using a similar method. Plasmid pBBR-MNX1–AS was constructed as follows. First, the gene set of *MNX1* and *AS* was PCR-amplified using pk18-MNX1–AS as the template. The amplified gene set was cloned into pBBR1MCS digested with *Xho*I and *Xba*I under the control of P_lca_ promoter. Other candidate genes were similarly cloned into pBBR1MCS, individually. The corresponding accession numbers of nucleotide sequence data were shown in Additional file 1, Table S2.

### Enzymatic assays of *MNX1* and *AS* expressed in chromosome

To evaluate *MNX1* and *AS* efficiency expressed in the chromosome, *P*. *chlororaphis* P3-Ar0 with *MNX1* and *AS* expressed in the *phzA* and *phzB* locus was activated in KB medium for 12 h at 28 °C. The overnight culture was then cultivated in triplicate erlenmeyer shake-flasks containing 60 mL KB liquid medium at the initial OD_600_ 0.02. After culturing for 12 h, the substrate 4-HBA was supplemented into the fermentation medium at the final concentration of 0, 0.5, 0.8, 1, 1.5, and 2 g/L. Before feeding 4-HBA, 4-HBA was dissolved in hot water to achieve a higher concentration and sterilized by filtration. After 24–72 h culturing at 28 °C and 200 rpm, samples were collected for the measurement of 4-HBA and arbutin by high-performance liquid chromatography (HPLC) every 12 h.

### Feeding experiments

The strain P3-Ar5 with the arbutin biosynthesis genes expressed on phenazine biosynthesis cluster under the control of *P*_*phz*_ was used for batch and fed-batch fermentation. It was activated in KBG medium at 28 °C for 24 h, and the overnight culture was inoculated in 60 mL KBG liquid medium at the initial OD_600_ 0.02. Different feeding strategies were carried out as follows. When feeding glycerol or glucose, 2.5 mL 432 g/L glycerol or 2.5 mL 216 g/L glucose was added to culture per 24 h. When co-feeding glycerol and glucose, 2.5 mL mixture of glycerol and glucose were used. After pre-culturing for 24 h, 2.5 mL mixture of glucose (216 g/L) and 4-HBA was supplemented into the cultures at the final concentration of glucose 9 g/L, 4-HBA 0.8 g/L per 24 h, while keeping the culture volume constant. The mixture of glucose and 4-HBA was dissolved in hot water to improve the solubility for 4-HBA, and sterilized by filtration. Samples were collected for 4-HBA and arbutin measurement.

### HPLC and UPLC-Q/TOF MS analysis

Metabolites of 4-HBA, quinol, and arbutin in the culture supernatant were analyzed using HPLC (Agilent Technologies 1260 Infinity) with a C18 reversed-phase column (5 μm, 4.6 × 12.5 mm). The mobile phase A was water containing 1‰ formic acid, and the mobile phase B was methanol containing 1‰ formic acid. The separation of metabolites was carried out via gradient elution under the following conditions: 0–2 min, 95% A; 2–10 min, 95–85% A; 10–15 min, 95% A, with a constant flow rate of 1 mL/min. The product concentrations were determined using an ultraviolet absorbance detector at 220 nm for arbutin and quinol, and 260 nm for 4-HBA. The concentrations of glycerol and glucose in the culture supernatant were quantified by HPLC equipped with an Aminex HPX-87H lon Exclusion Column (300 × 7.8 mm, Bio-Rad, Hercules, CA) and a refractive index (RID-10A) detector. The separation was carried out at 60 °C at a flow rate of 0.6 mL/min with 5 mM H_2_SO_4_ as the mobile phase. In all cases, external standards were prepared to provide calibrations for concentration determination.

The samples were analyzed by the ultra-high performance liquid chromatography-quadrupole time-of-flight mass spectrometry (UPLC-Q/TOF MS) as reported earlier [17, 36]. Mobile phase A (water containing 1‰ formic acid) and B (methanol containing 1‰ formic acid) were used as the following gradient: 0–2 min, 95% A; 2–10 min, 95–85% A; 10–15 min, 95% A, with a constant flow rate of 0.2 mL/min.

## Additional file

**Additional file 1.** Additional Figure S1–S4 and Tables S1–S2. **Table S1.** Main primers designed and used in this study. **Table S2.** The corresponding accession numbers of nucleotide sequence data. **Figure S1.** Identification of arbutin by LC-MC analyses. **Figure S2.** The change of pH value of P3-Ar5 cultures in KBG medium. **Figure S3.** The arbutin titer of the plasmid-free strain P3-Ar5 after culturing for 1–30 generations in KBG medium.

## Abbreviations

4-HBA: 4-hydroxybenzoic acid
DCW: dry cell weight
E4P: erythrose 4-phosphate
PEP: phosphoenolpyruvate
TAC cycle: tricarboxylic acid cycle
UDPG: uridine diphosphate glucose
HQ: hydroquinone
