# Supplementary material for: Enhanced biosynthesis of arbutin by engineering shikimate pathway in Pseudomonas chlororaphis P3
Source: Microb Cell Fact. 2018 Nov 10;17:174. doi: 10.1186/s12934-018-1022-8 (PMC6230248; doi:10.1186/s12934-018-1022-8)
Supplement: Supplementary file 1 — Additional file 1. Additional Figure S1–S4 and Tables S1–S2. Table S1. Main primers designed and used in this study. Table S2. The corresponding accession numbers of nucleotide sequence data. Figure S1. Identification of arbutin by LC-MC analyses. Figure S2. The change of pH value of P3-Ar5 cultures in KBG medium. Figure S3. The arbutin titer of the plasmid-free strain P3-Ar5 after culturing for 1–30 generations in KBG medium. [file 12934_2018_1022_MOESM1_ESM.docx]

**Enhanced biosynthesis of arbutin by engineering shikimate pathway in *Pseudomonas* *chlororaphis* P3**

Songwei Wang^1^, Cong Fu^1^, Muhammad Bilal^1^, Hongbo Hu^1,2^, Wei Wang^1^, Xuehong Zhang^1^*

^1^State Key Laboratory of Microbial Metabolism, School of Life Sciences and Biotechnology, Shanghai Jiao Tong University, Shanghai, 200240, China

^2^National Experimental Teaching Center for Life Sciences and Biotechnology, Shanghai Jiao Tong University, Shanghai, 200240, China

***Corresponding author:** xuehzhang@sjtu.edu.cn (Xuehong Zhang); **Tel: +86-21-3420-6742 Fax: +86-21-3420-6791**

**Table S1.** Main primers designed and used in this study

| **Primer** | **Sequence (5’-3’)** |
| --- | --- |
| For gene deletion | ***pykA* deletion** |
| pykA-1F | catgattacgaattcTGCGCGAAGGGTAATGCCCCAGTTG |
| pykA-1R | GCAAAGACTCCTGAGTTCAAGCGCA |
| pykA-2F | TGCGCTTGAACTCAGGAGTCTTTGCGCCACCTGACGCAACAATAAAGAGC |
| pykA-2R | GACTCTAGAGGATCCCCGCGCCTGCTCGGGCAGGCCAAGG |
| For gene overexpression | ***P_phz_-XanB2*-*phzC*, overexpression on gene *pykA* locus** |
| G-UF | catgattacgaattcCAACTGGGGCATTACCCTTCGCGCA |
| G-UR | GCAAAGACTCCTGAGTTCAAGCGCA |
| G-PF | TGCGCTTGAACTCAGGAGTCTTTGCTTTGAGCACCACTAAAGTTGAAAAC |
| G-PR | GGCGGCATCCTCCTTAGTTGGGCTG |
| G-XF | CAGCCCAACTAAGGAGGATGCCGCCATGACCGCCCCGACCCTGCAGCCGA |
| G-XR | TCAGCCGTGGACGCCGTCGATTTCC |
| G-CF | GGAAATCGACGGCGTCCACGGCTGATTCTCTCGTGAGAGTGATCGCATCA |
| G-CR | TCAAAAGGAGGCAAGGGTTGAGGAG |
| G-DF | CTCCTCAACCCTTGCCTCCTTTTGAGCCACCTGACGCAACAATAAAGAGC |
| G-DR | GACTCTAGAGGATCCCCGCGCCTGCTCGGGCAGGCCAAGG |
| For gene substitution | ***P_phz_-XanB2* inserted to *phzD* locus** |
| XD-1F | catgattacgaattcGGACCAGTTGCTAAGCCTGTGTGAA |
| XD-1R | GGCGACAAACTCCAGTCAAAAGGA |
| XD-2F | TCCTTTTGACTGGAGTTTGTCGCCTTTGAGCACCACTAAAGTTGAAAACAGG |
| XD-2R | GGCGGCATCCTCCTTAGTTGGG |
| XD-3F | CCCAACTAAGGAGGATGCCGCCATGACCGCGCCAACGCTC |
| XD-3R | TCAGCCGTGCACGCCGTC |
| XD-4F | GACGGCGTGCACGGCTGAATGAACCAAGCCGCAGCCC |
| XD-4R | GACTCTAGAGGATCCAAAGAACGACAGCGCACTGGC |
| For pathway construction | ***MNX1-AS,* substitute *phzA*, *phzB*** |
| Ar-1F | catgattacgaattcTGTCCATGGTTCGCGCAAAGA |
| Ar-1R | GGCGGCATCCTCCTTAGTTGG |
| Ar-2F | CCAACTAAGGAGGATGCCGCCATGGCCGTGCAAGCCCCCTCGAAGA |
| Ar-2R | TCAGCCGCTGGCGCTCAGCGGCGCG |
| Ar-3F | CGCGCCGCTGAGCGCCAGCGGCTGA*AATTCGGCACGAGCTGCAACGAAAGC*ATGGAACACACCCCGCACATCGCCA |
| Ar-3R | TCAGGTCGACGAGATTTTGTTTTCC |
| Ar-4F | GGAAAACAAAATCTCGTCGACCTGATTCTCTCGTGAGAGTGATCGCATCA |
| Ar-4R | GACTCTAGAGGATCCGCTATAGCCTCGAACCAGGCGTT |

15~20 bases of homology sequence for In-fusion kit are underlined

**Table S2.** The accession number of genes used in this work was deposited in GenBank of NCBI.

| **Gene** | **Locus Tag** | **Reference Sequence Accession** |
| --- | --- | --- |
| *pykA* | M217_RS0111030 | NZ_ATBG01000023 |
| *rpeA* | M217_RS0125400 | NZ_ATBG01000041 |
| *rsmE* | M217_RS0101235 | NZ_ATBG01000008 |
| *lon1* | M217_RS0104945 | NZ_ATBG01000015 |
| *lon2* | M217_RS0111675 | NZ_ATBG01000024 |
| *pobA* | M217_RS0111140 | NZ_ATBG01000023 |
| *phzE* | M217_RS0112900 | NZ_ATBG01000024 |
| *phzC* | M217_RS0112890 | NZ_ATBG01000024 |
| *aroB* | M217_RS0123005 | NZ_ATBG01000040 |
| *aroD* | M217_RS0120595 | NZ_ATBG01000037 |
| *aroE* | M217_RS0116465 | NZ_ATBG01000028 |
| *ppsA* | M217_RS0102095 | NZ_ATBG01000009 |
| *tktA* | M217_RS0118875 | NZ_ATBG01000029 |
| *XanB2* |  | CCE40241.1 |
| *AS* |  | AJ310148.1 |
| *MNX1* |  | [CCE40241.1](https://www.ncbi.nlm.nih.gov/protein/CCE40241.1?report=genbank&log$=prottop&blast_rank=1&RID=F9RJ40MU015) |


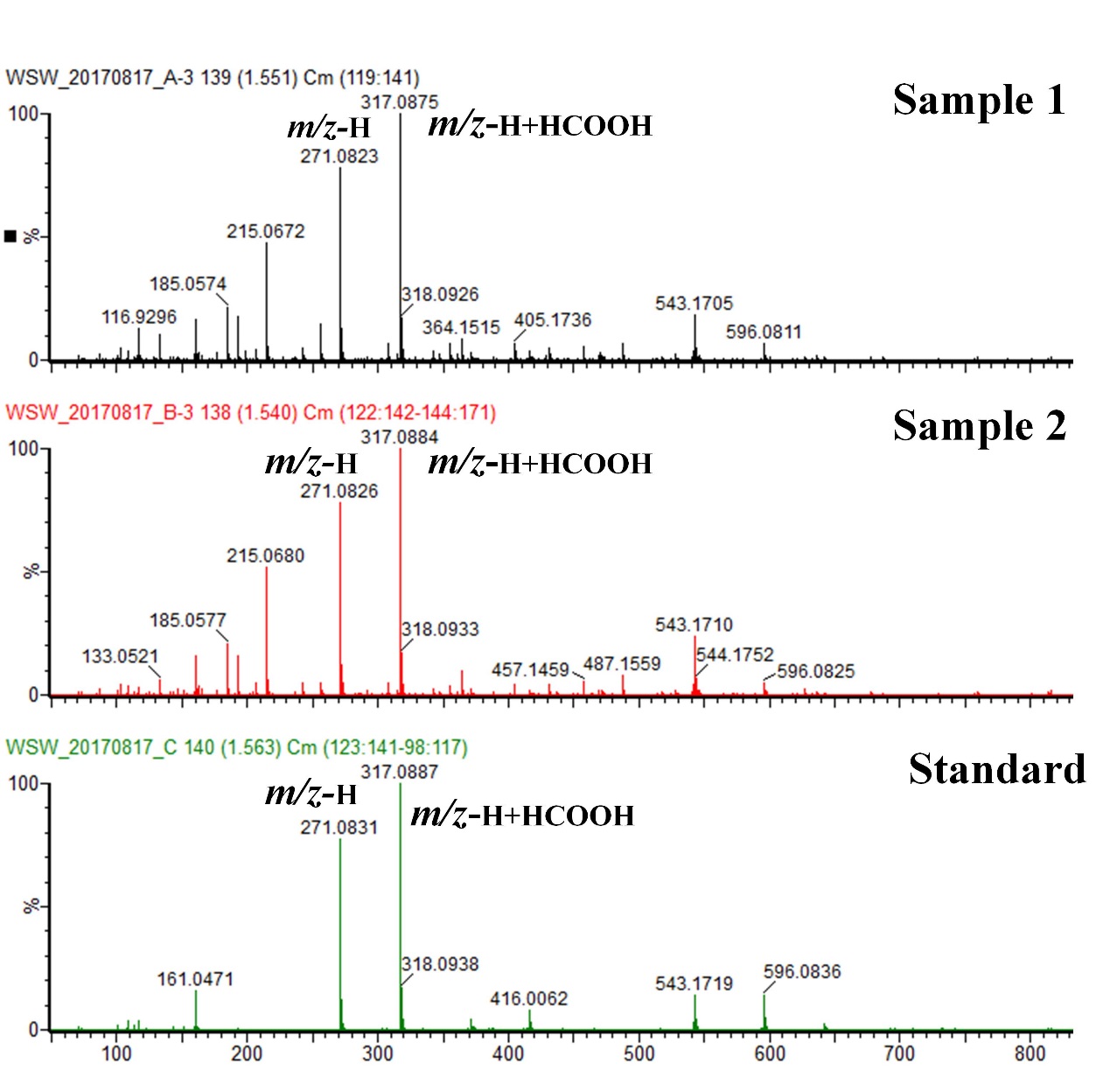


**Figure S1.** Identification of arbutin by LC-MC analyses

Sample 1, Arbutin produced by P3-Ar0 via feeding 4-HBA; Sample 2, Arbutin produced by culture of P3-Ar1; Standard, standard of arbutin.

**Figure S2.** The change of pH value of P3-Ar5 cultures when fermented in KBG medium.

**Figure S3.** The arbutin titer of the plasmid-free strain P3-Ar5 after culturing for 1-30 generations in KBG medium.
